# Supplementary figures and images for: The yeast RNA methylation complex consists of conserved yet reconfigured components with m6A-dependent and independent roles
Source: eLife. 2023 Jul 25;12:RP87860. doi: 10.7554/eLife.87860 (PMC10393049; doi:10.7554/eLife.87860)

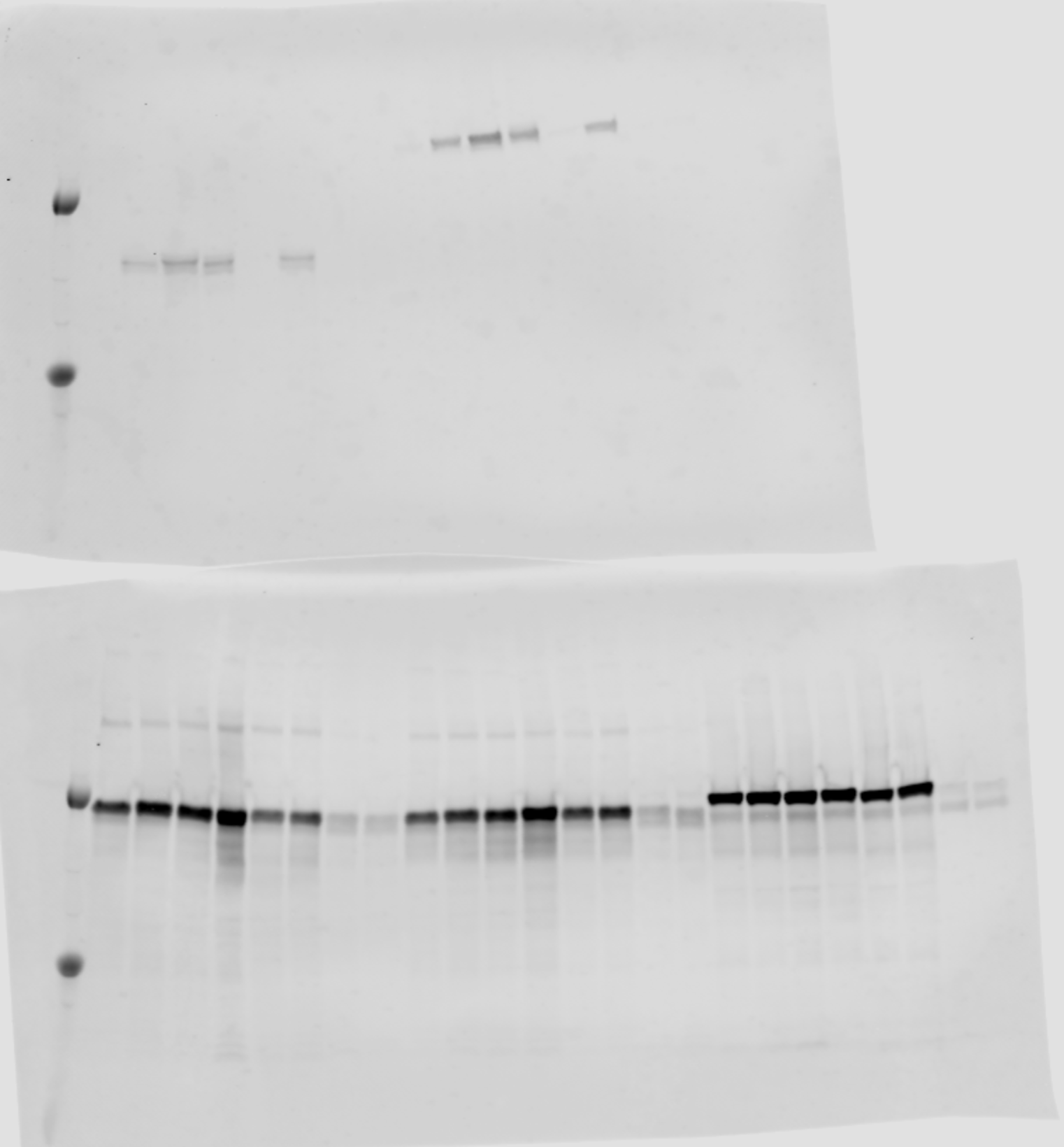

Supplement: Figure 2—figure supplement 1—source data 1. [file elife-87860-fig2-figsupp1-data1.zip › Figure 2 - figure supplement 1 - source data 1.png]

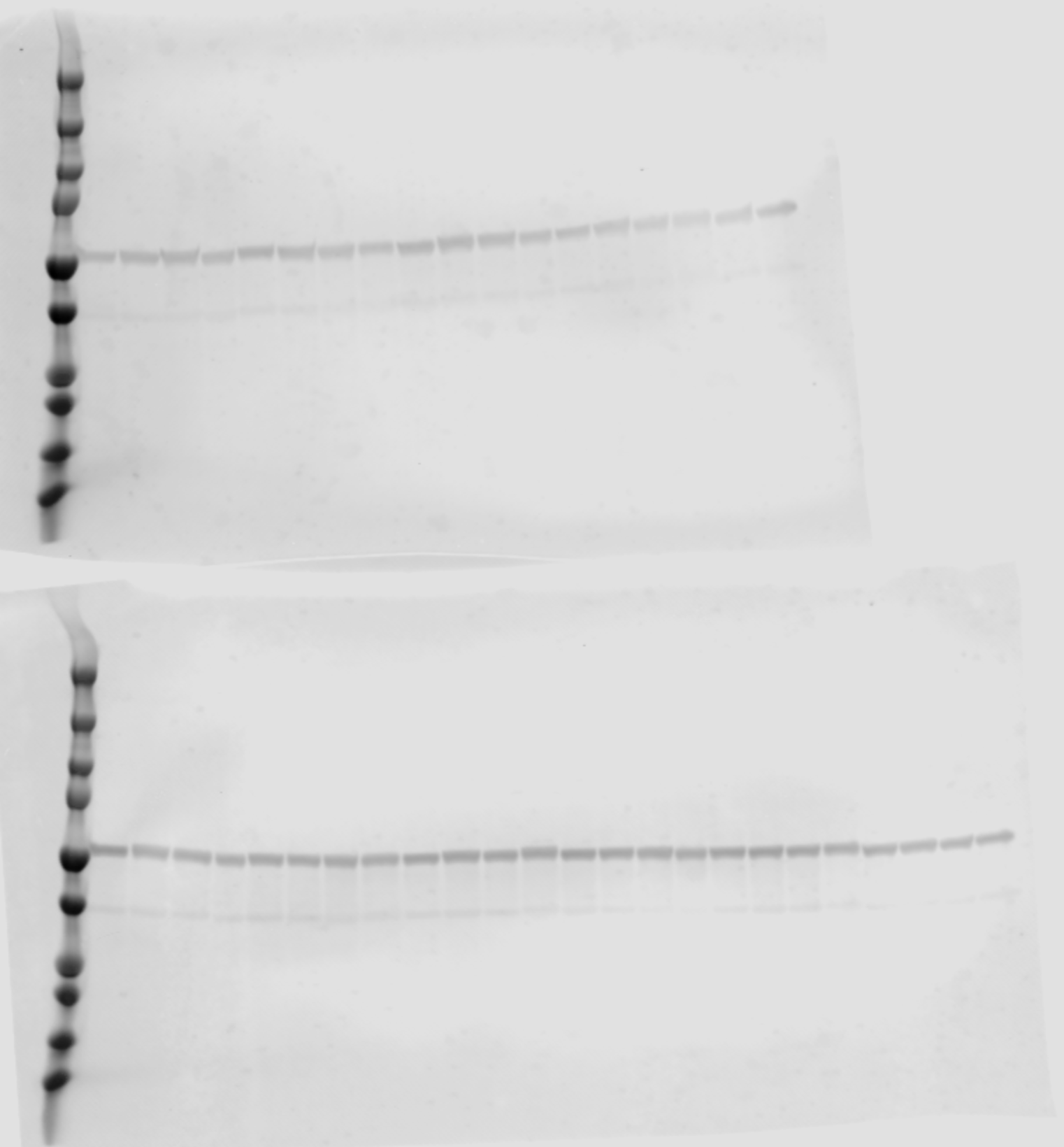

Supplement: Figure 2—figure supplement 1—source data 2. [file elife-87860-fig2-figsupp1-data2.zip › Figure 2 - figure supplement 1 - source data 2.png]

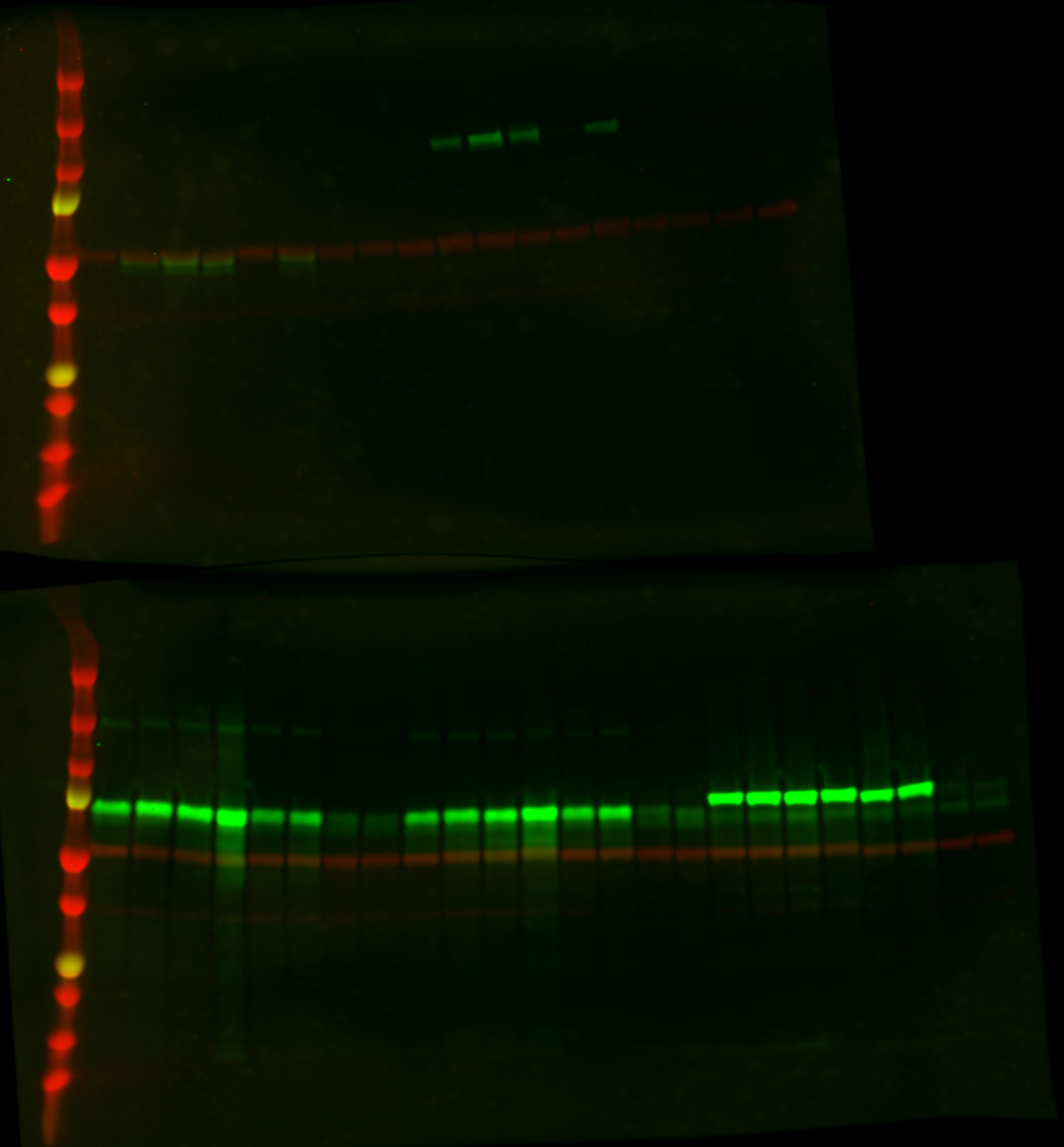

Supplement: Figure 2—figure supplement 1—source data 3. [file elife-87860-fig2-figsupp1-data3.zip › Figure 2 - figure supplement 1 - source data 3.png]

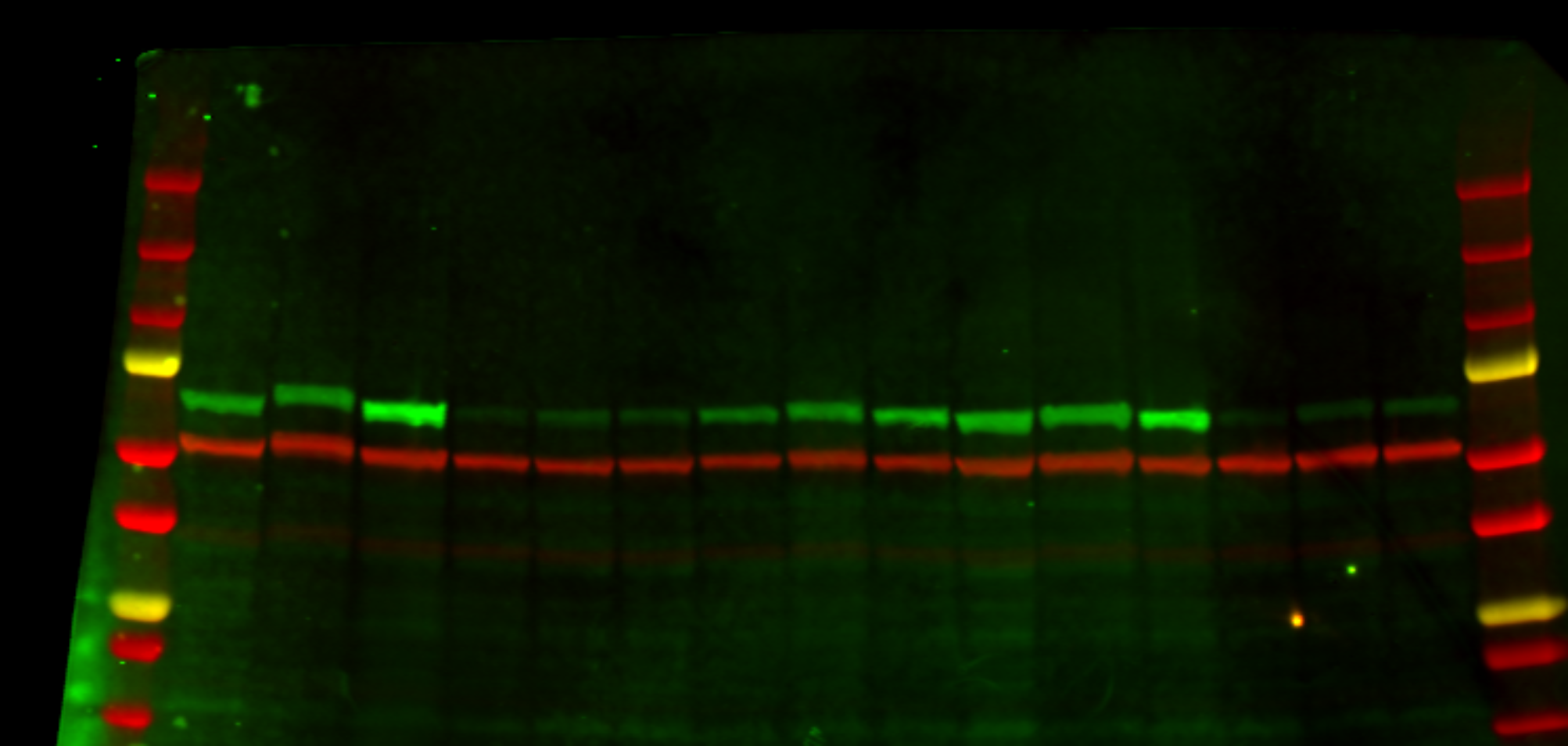

Supplement: Figure 4—source data 1. [file elife-87860-fig4-data1.zip › Figure 4A - source data 1.tif]

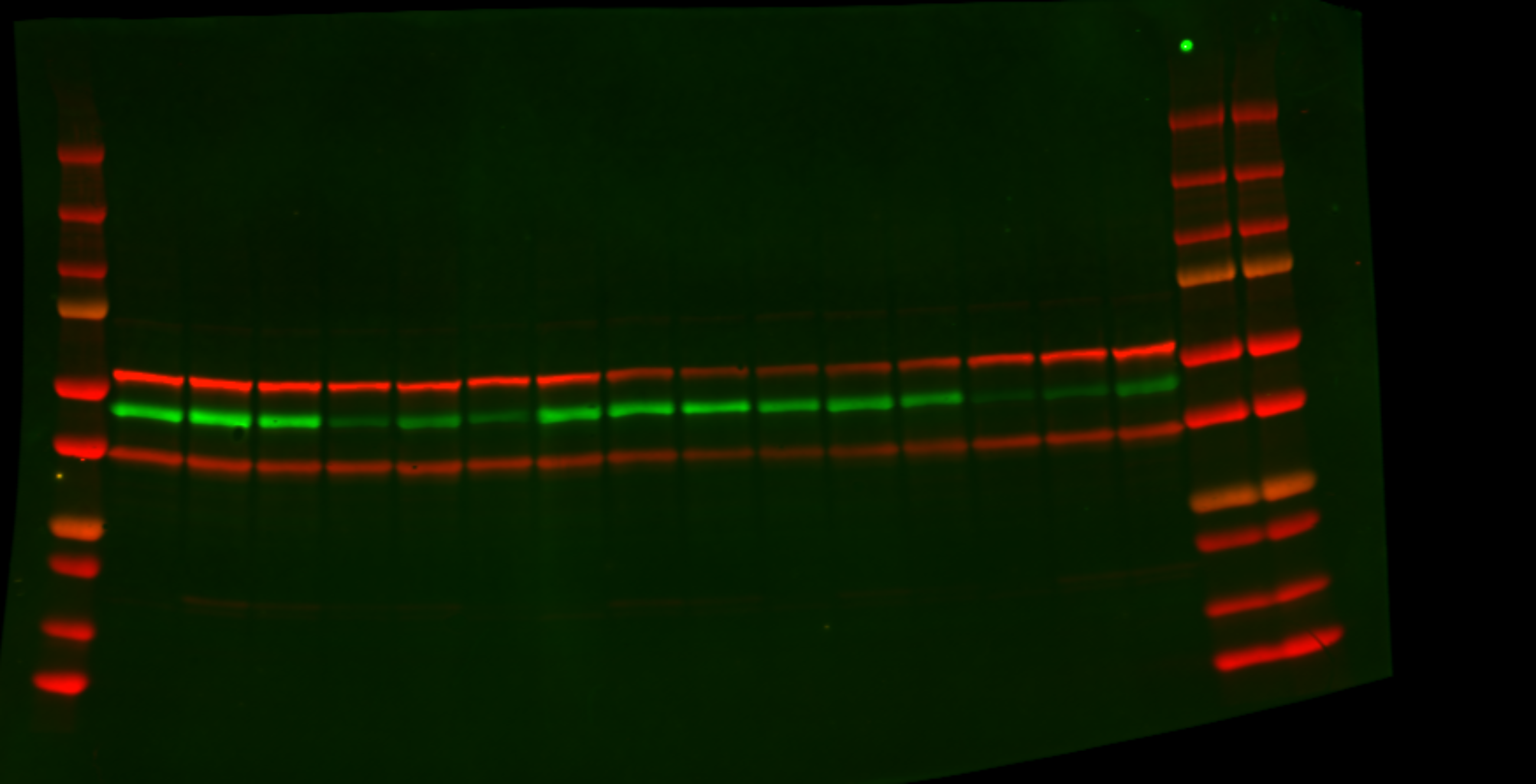

Supplement: Figure 4—source data 2. [file elife-87860-fig4-data2.zip › Figure 4A - source data 2.tif]

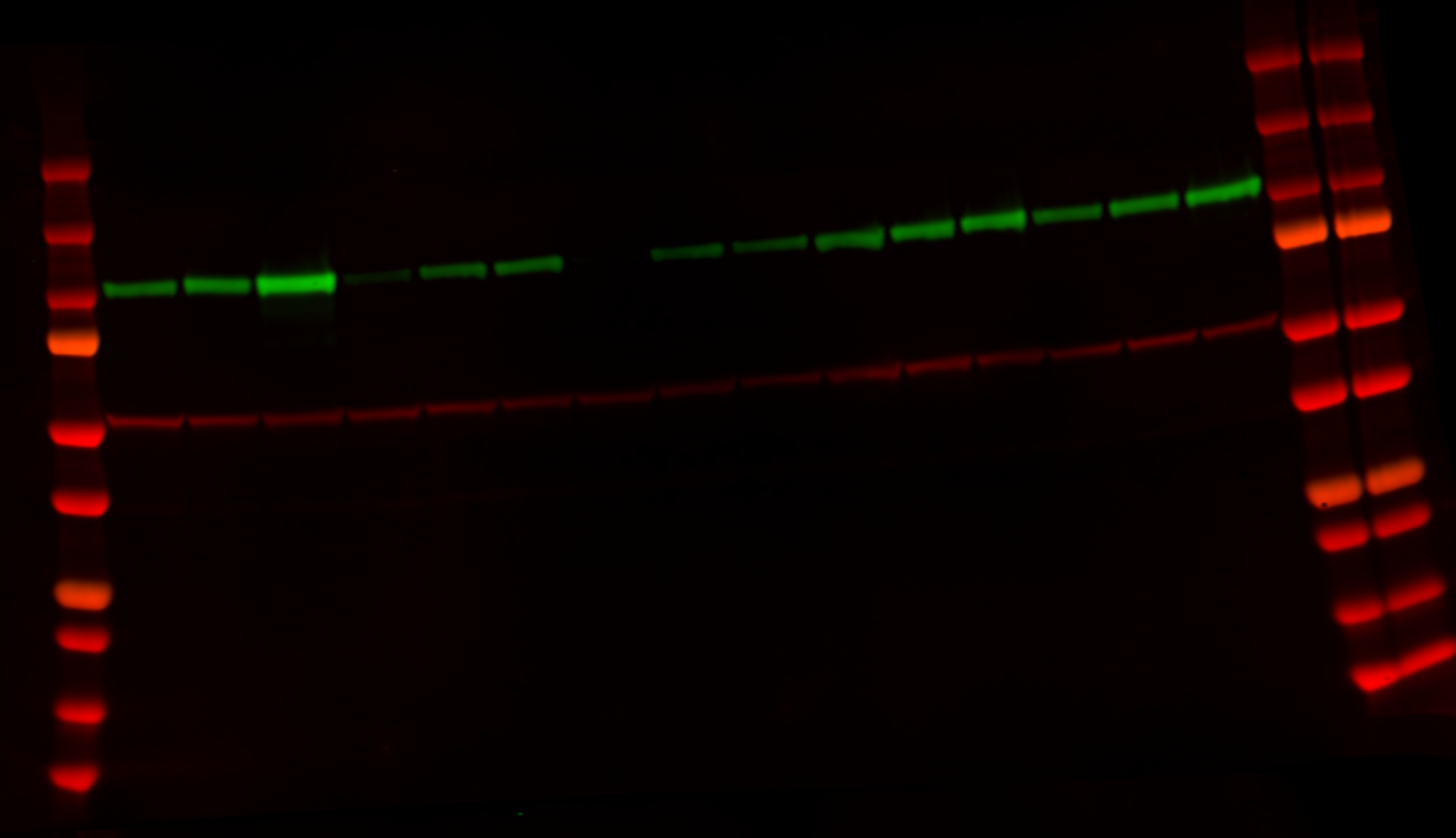

Supplement: Figure 4—source data 3. [file elife-87860-fig4-data3.zip › Figure 4A - source data 3.tif]

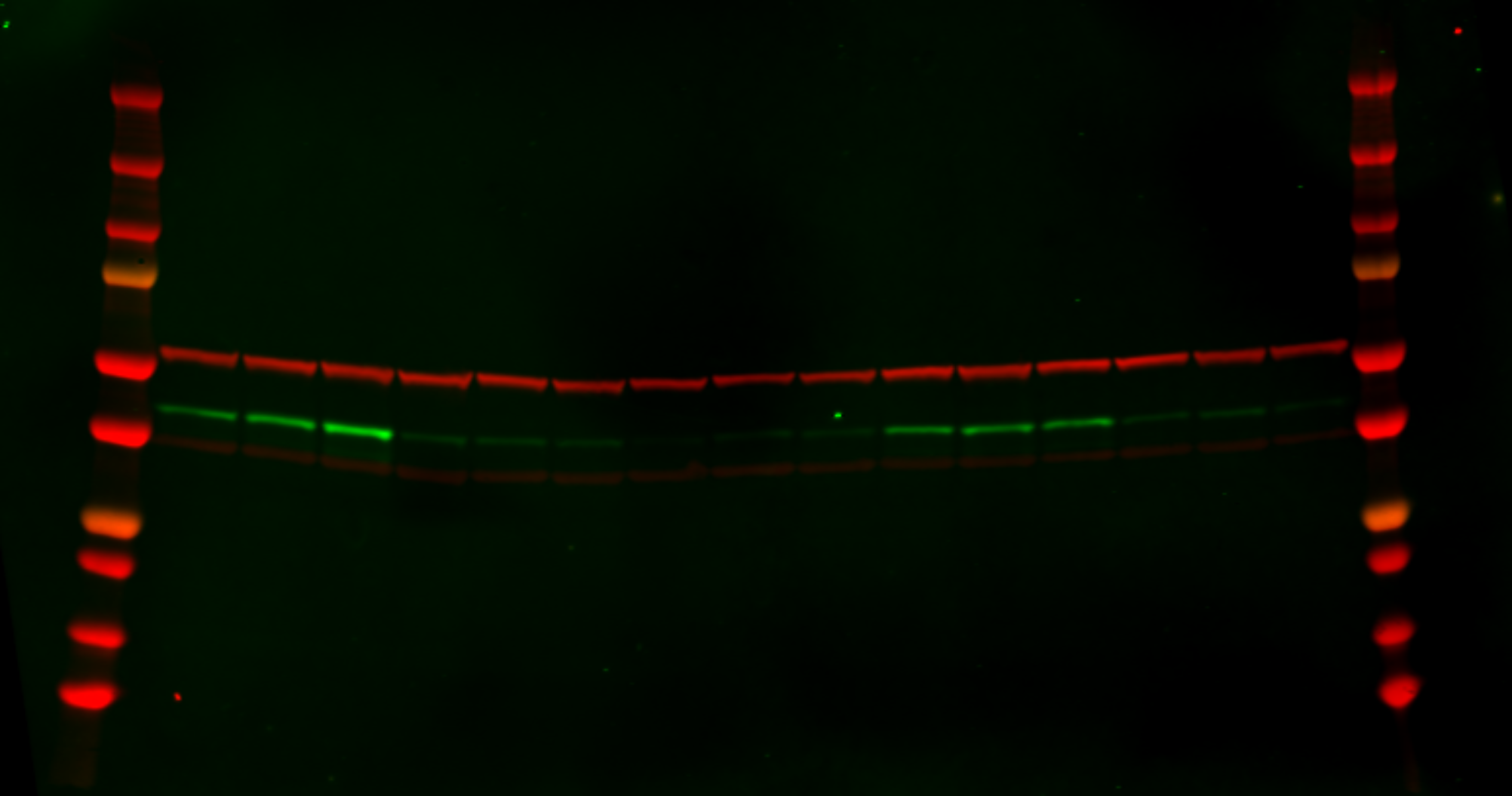

Supplement: Figure 4—source data 4. [file elife-87860-fig4-data4.zip › Figure 4A - source data 4.tif]

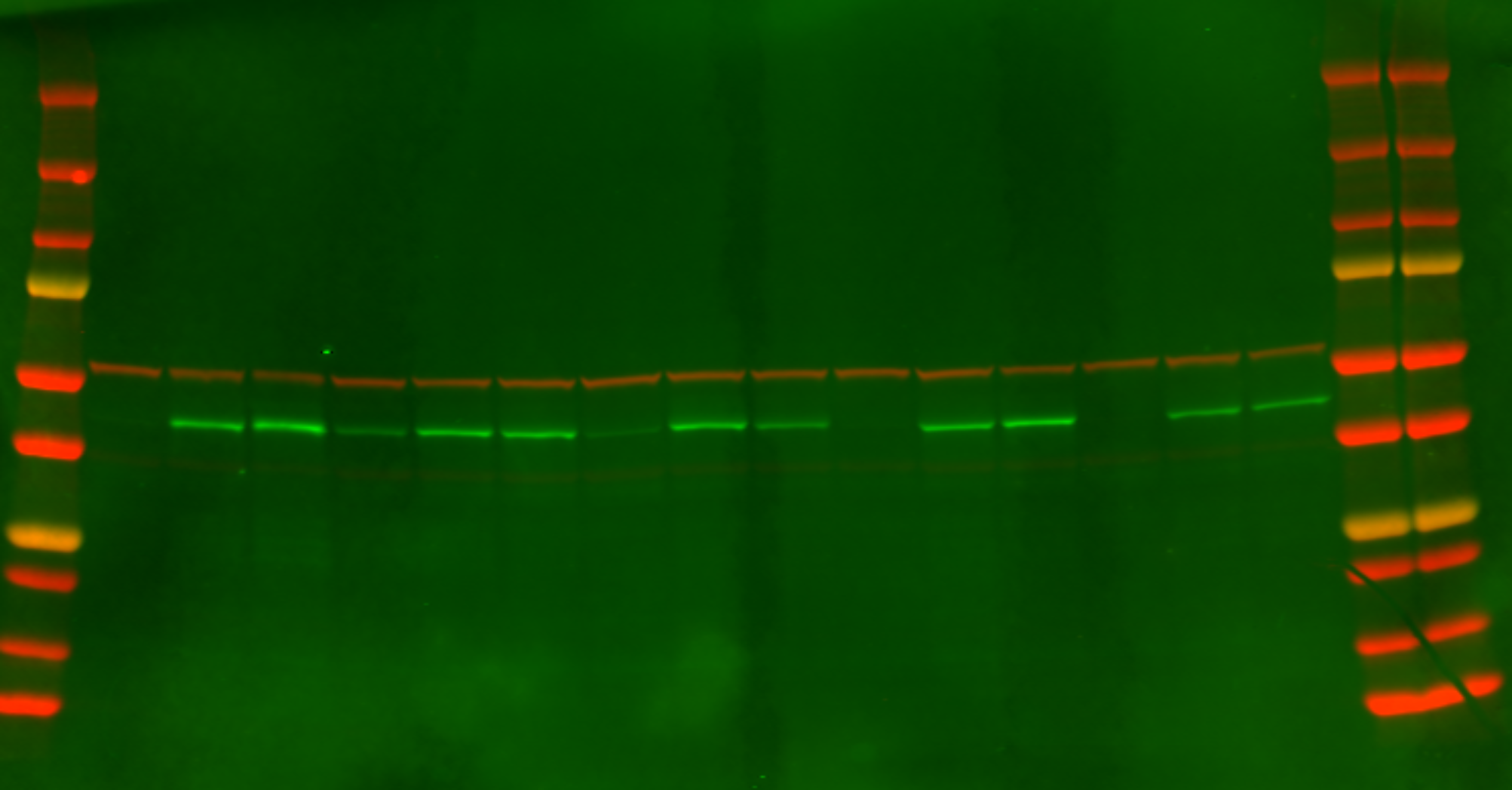

Supplement: Figure 4—source data 5. [file elife-87860-fig4-data5.zip › Figure 4A - source data 5.tif]

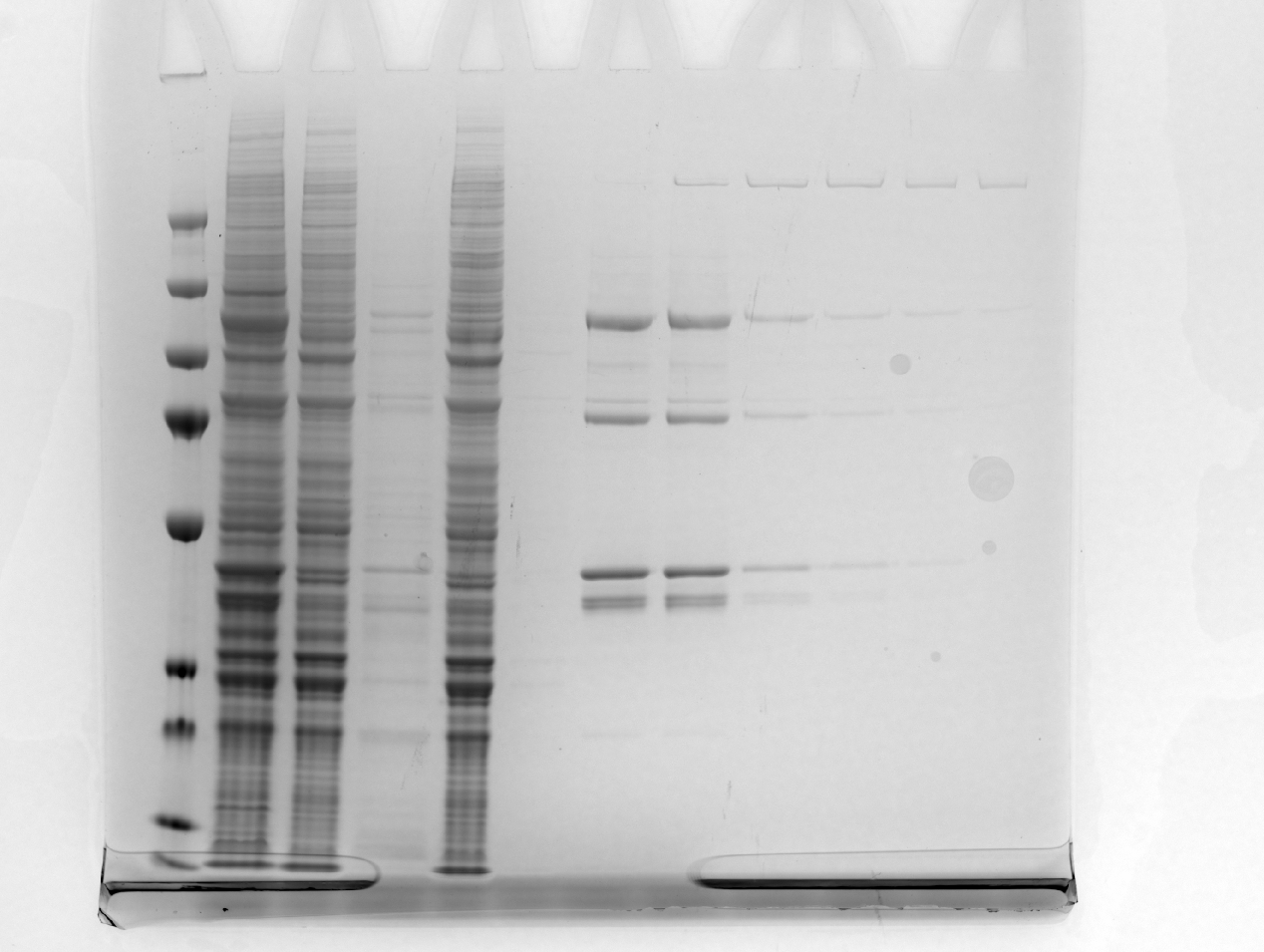

Supplement: Figure 4—source data 6. [file elife-87860-fig4-data6.zip › Figure 4E - source data 1.tif]

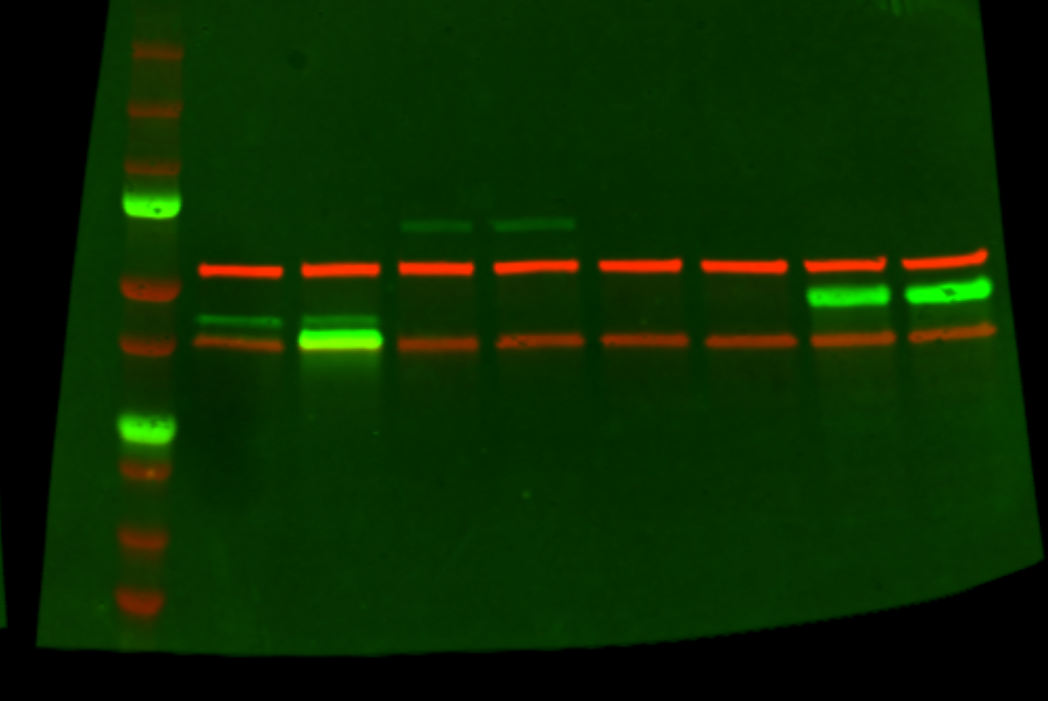

Supplement: Figure 6—source data 1. [file elife-87860-fig6-data1.zip › Figure 6A - source data 1.tif]

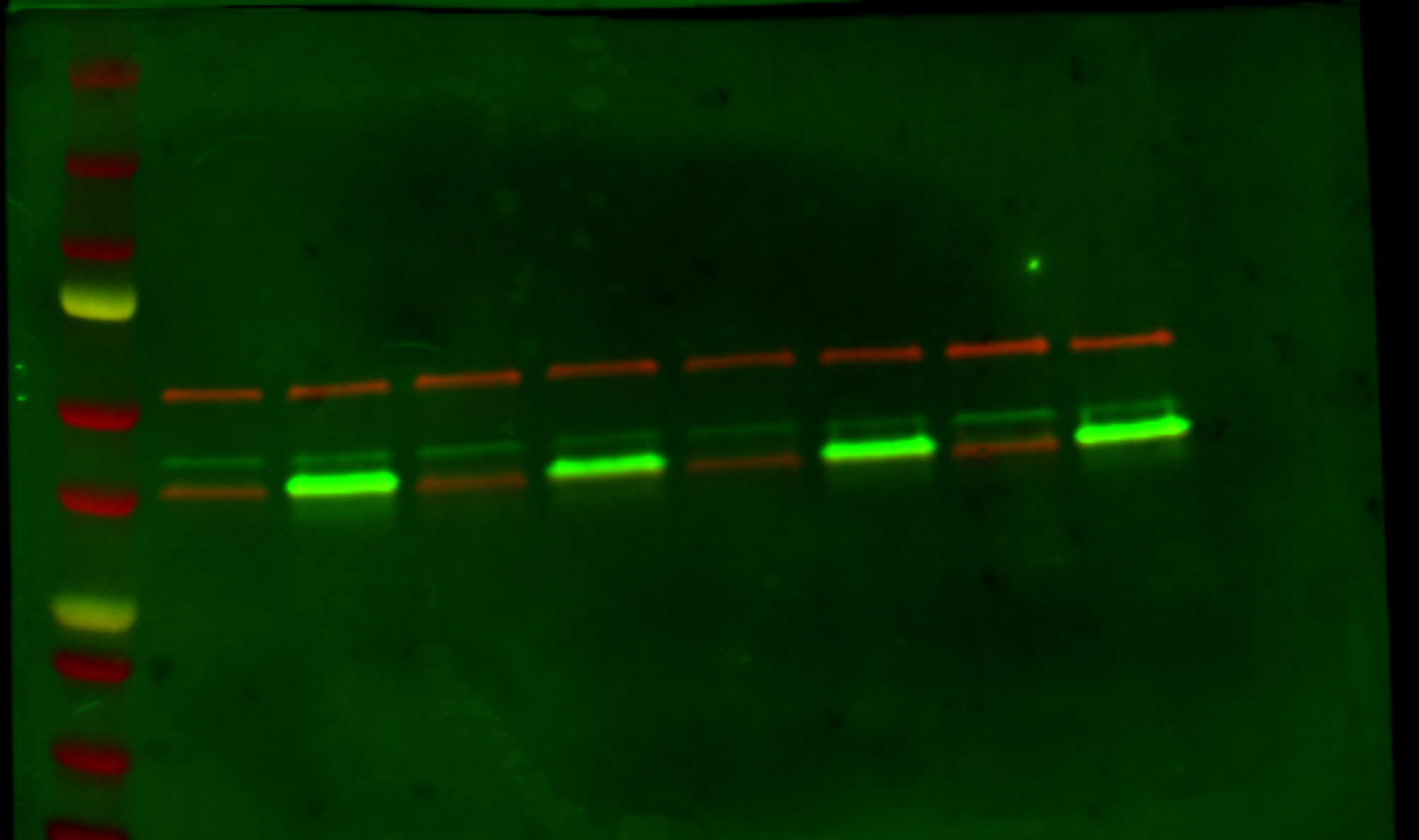

Supplement: Figure 6—source data 2. [file elife-87860-fig6-data2.zip › Figure 6B - source data 1.tif]

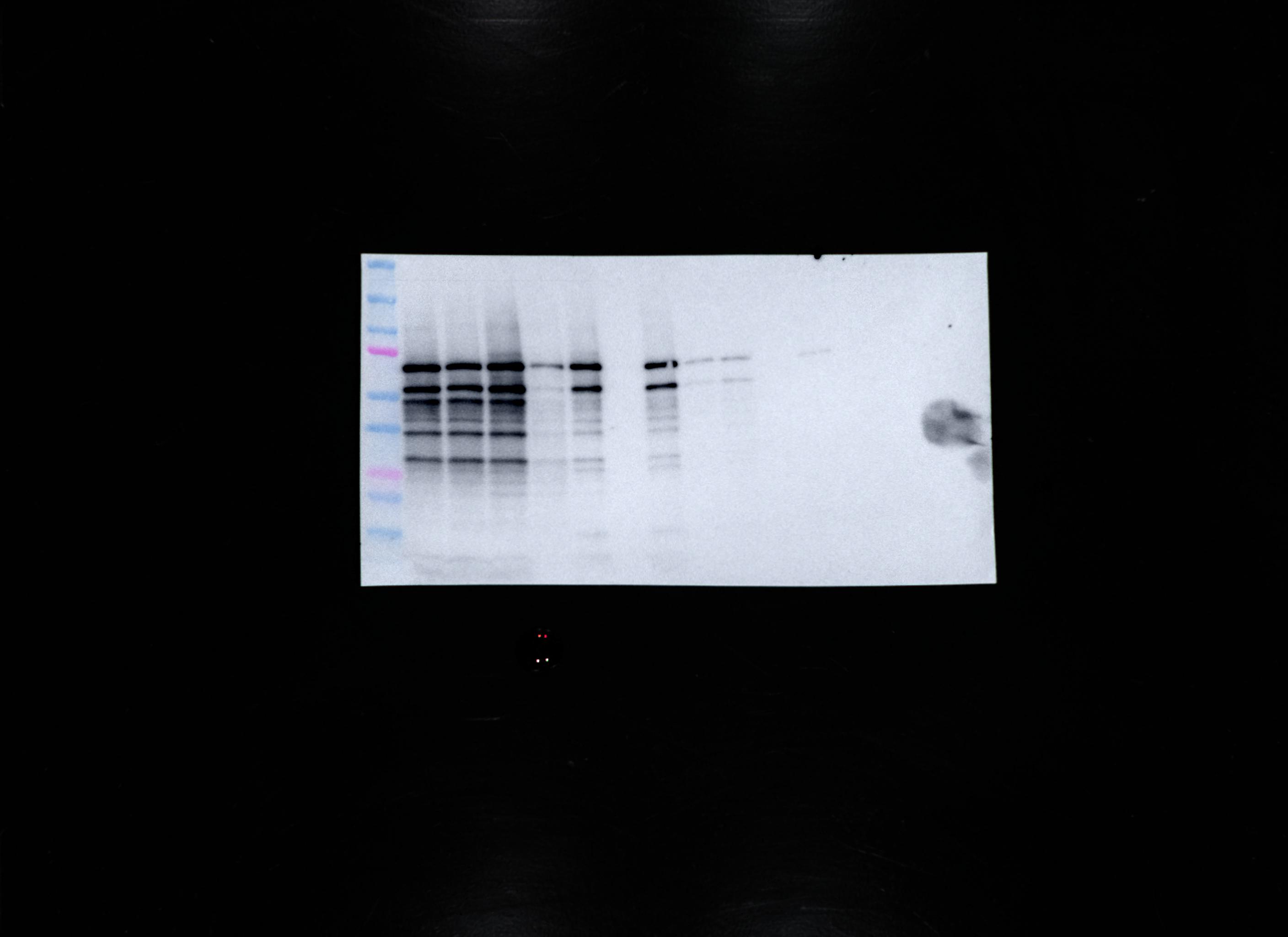

Supplement: Figure 6—source data 4. [file elife-87860-fig6-data4.zip › Figure 6J - source data 1.jpg]

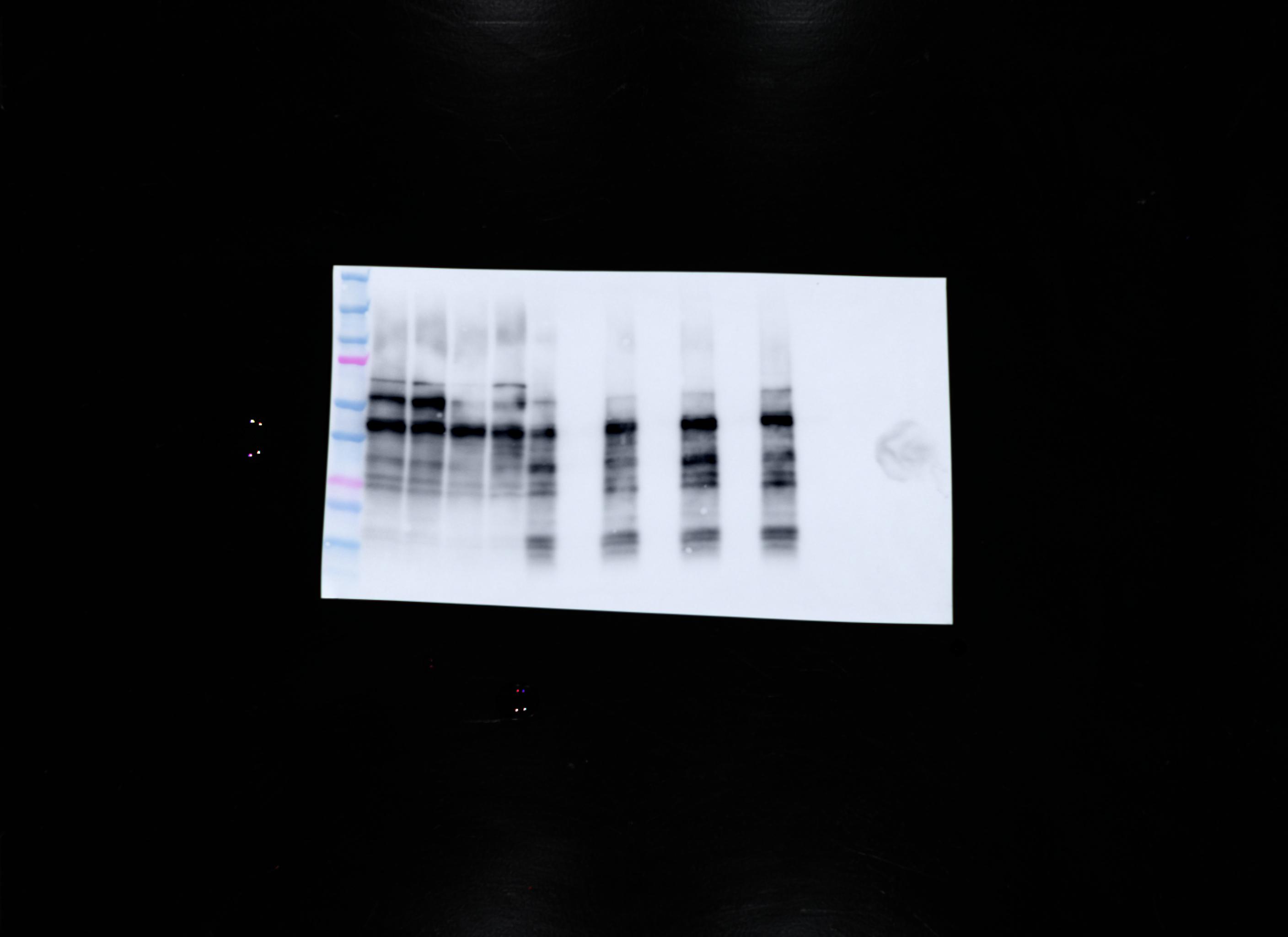

Supplement: Figure 6—source data 5. [file elife-87860-fig6-data5.zip › Figure 6J - source data 2.jpg]

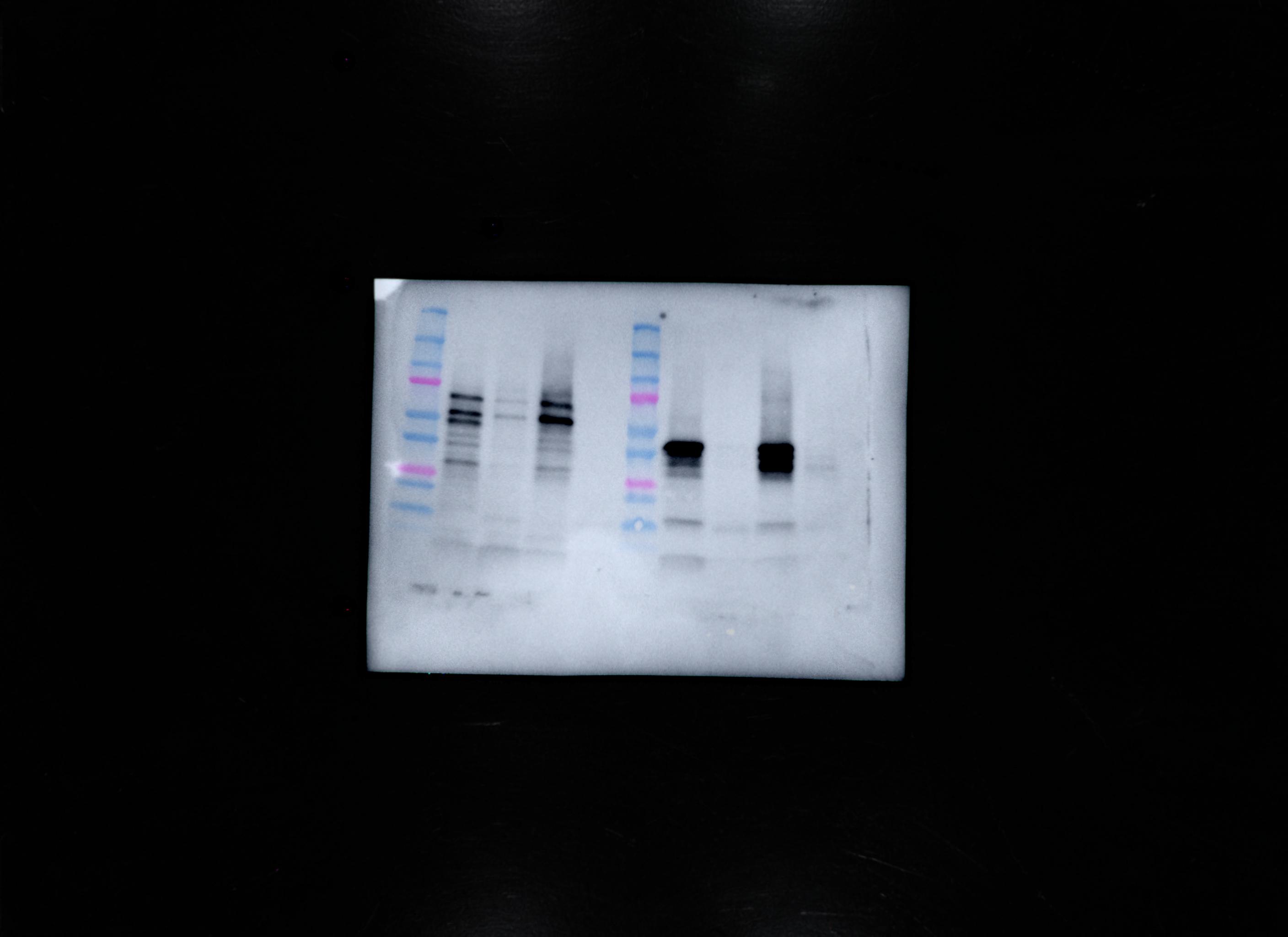

Supplement: Figure 6—figure supplement 1—source data 1. [file elife-87860-fig6-figsupp1-data1.zip › Figure 6 - figure supplement 1F - source data 1.jpg]

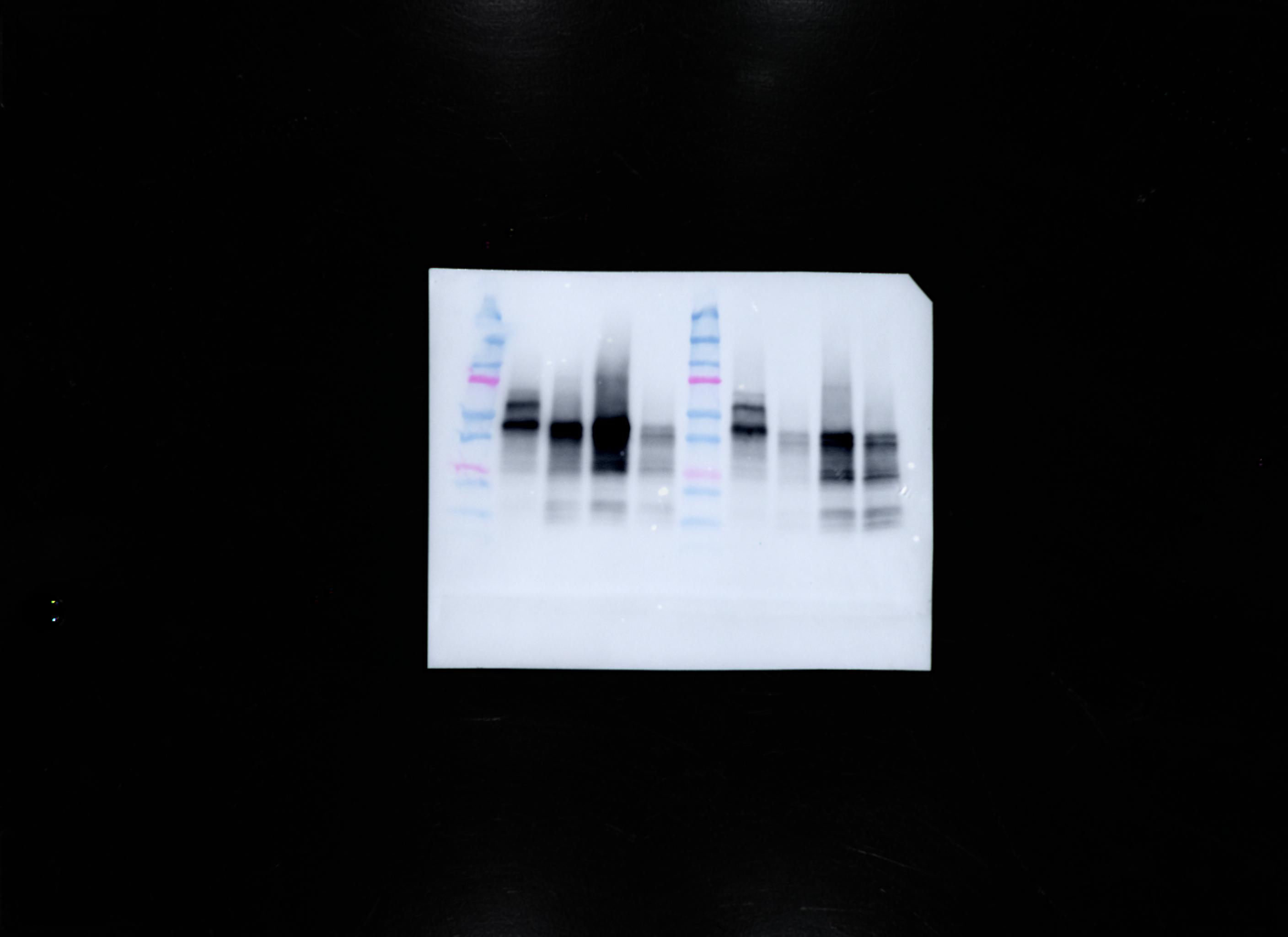

Supplement: Figure 6—figure supplement 1—source data 2. [file elife-87860-fig6-figsupp1-data2.zip › Figure 6 - figure supplement 1F - source data 2.jpg]

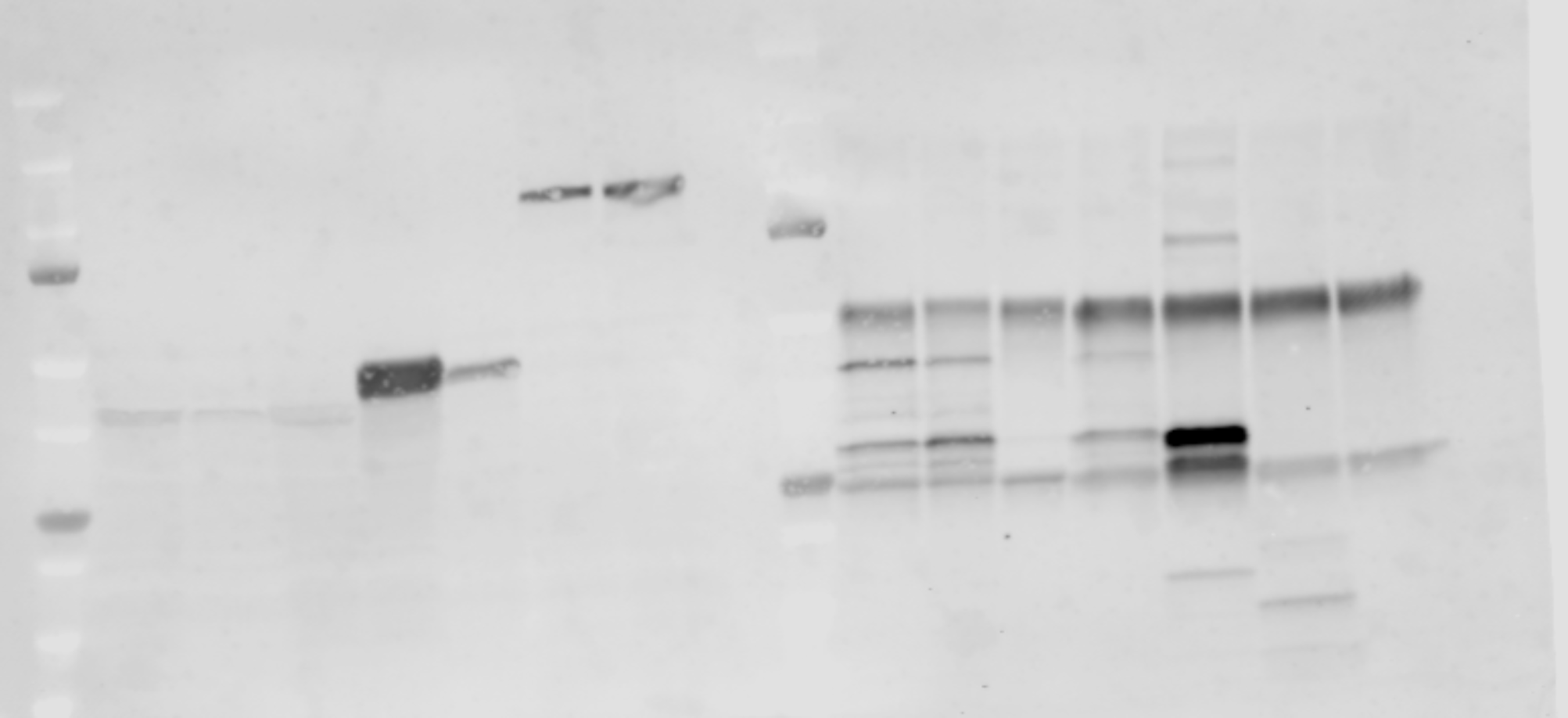

Supplement: Figure 6—figure supplement 1—source data 3. [file elife-87860-fig6-figsupp1-data3.zip › Figure 6 - figure supplement 1G - source data 1.tif]
