# Supplementary material for: The yeast RNA methylation complex consists of conserved yet reconfigured components with m6A-dependent and independent roles
Source: eLife. 2023 Jul 25;12:RP87860. doi: 10.7554/eLife.87860 (PMC10393049; doi:10.7554/eLife.87860)
Supplement: Supplementary file 4. [file elife-87860-supp4.docx]

**Oligo nucleotide sequences used.**

**Name Sequence**

IE34 pAGA1 ChIP qPCR fw AGGGTACCTGTCACATATATTCTCA

IE35 pAGA1 ChIP qPCR rv ATTATGTTACAGCCGCGTTTTG

HMR1F_RT acgatccccgtccaagttatg

HMR1R_RT cttcaaaggagtcttaatttccctg
