# Supplementary material for: The yeast RNA methylation complex consists of conserved yet reconfigured components with m6A-dependent and independent roles
Source: eLife. 2023 Jul 25;12:RP87860. doi: 10.7554/eLife.87860 (PMC10393049; doi:10.7554/eLife.87860)
Supplement: Supplementary file 5. [file elife-87860-supp5.docx]

**Plasmids used.**

**nr name**

p227 pWG444 NAT

p17 pFA6a-kanMX6

p147 pFA6a.URA3Mx6

p78 pFA6a-TEV-ProA-KanMX6

p255 pL264 KAN -3v5

p386 pCA13-mNeongreen(Yeast Optimized)-NAT

p491 3Pk-miniAID-kanMX

p782 Ime4-v5 TRP wt integration plasmid

p783 Ime4-v5 TRP catalytic dead integration plasmid
